# Prostate Tumor-Derived Exosomes Down-Regulate NKG2D Expression on Natural Killer Cells and CD8^+^ T Cells: Mechanism of Immune Evasion

**DOI:** 10.1371/journal.pone.0108925

**Published:** 2014-09-30

**Authors:** Marie Lundholm, Mona Schröder, Olga Nagaeva, Vladimir Baranov, Anders Widmark, Lucia Mincheva-Nilsson, Pernilla Wikström

**Affiliations:** 1 Department of Medical Biosciences, Pathology, Umeå University, Umeå, Sweden; 2 Department of Clinical Microbiology, Clinical Immunology, Umeå University, Umeå, Sweden; 3 Department of Radiation Sciences, Oncology, Umeå University, Umeå, Sweden; Gustave Roussy, France

## Abstract

Tumor-derived exosomes, which are nanometer-sized extracellular vesicles of endosomal origin, have emerged as promoters of tumor immune evasion but their role in prostate cancer (PC) progression is poorly understood. In this study, we investigated the ability of prostate tumor-derived exosomes to downregulate NKG2D expression on natural killer (NK) and CD8^+^ T cells. NKG2D is an activating cytotoxicity receptor whose aberrant loss in cancer plays an important role in immune suppression. Using flow cytometry, we found that exosomes produced by human PC cells express ligands for NKG2D on their surface. The NKG2D ligand-expressing prostate tumor-derived exosomes selectively induced downregulation of NKG2D on NK and CD8^+^ T cells in a dose-dependent manner, leading to impaired cytotoxic function *in vitro*. Consistent with these findings, patients with castration-resistant PC (CRPC) showed a significant decrease in surface NKG2D expression on circulating NK and CD8^+^ T cells compared to healthy individuals. Tumor-derived exosomes are likely involved in this NKG2D downregulation, since incubation of healthy lymphocytes with exosomes isolated from serum or plasma of CRPC patients triggered downregulation of NKG2D expression in effector lymphocytes. These data suggest prostate tumor-derived exosomes as down-regulators of the NKG2D-mediated cytotoxic response in PC patients, thus promoting immune suppression and tumor escape.

## Introduction

Exosomes are small (30- to 100-nm) membrane vesicles of endocytic origin that are actively secreted from most cell types. They contain a variety of biologically active molecules such as proteins, mRNAs, and microRNAs reflecting the cell of origin, and they probably mediate a range of local and systematic functions [1]–[4]. Tumor cells produce large amounts of exosomes, which has been shown *in vitro* and by purification from plasma, ascites, and pleural effusions from cancer patients [5]–[8]. Tumor-released exosomes have been suggested to influence immune responses and possibly contribute to cancer progression [9], [10].

The NKG2D/NKG2DL system plays an important role in tumor immune surveillance [11]–[13]. The activating receptor NKG2D is expressed by a variety of immune cells, including NK cells, NKT cells, CD8^+^ T cells, and subsets of γδ^+^ T cells [14], [15]. Ligands for NKG2D, the MHC class I chain-related (MIC) proteins A and B, and the UL-16 binding proteins (ULBPs), are rarely expressed on healthy cells; instead, their expression is upregulated as a result of different kinds of cellular stress such as viral infections and malignant transformation [16]. NKG2D ligands are frequently overexpressed on a broad range of epithelial tumors, including prostate cancer (PC), making them highly susceptible to killing by NK cells and T cells [17]–[19]. On the other hand, it is known that tumors can escape the host immune system by secreting a soluble form of MIC (sMIC). sMIC binds to NKG2D and downregulates its expression, leading to loss of the NK/T cell activation trigger [20], [21]. Moreover, there is convincing evidence that exosomes derived from diverse cancer cell lines, including mesothelioma, breast and prostate cancer cells, express NKG2D ligands and thereby downregulate NKG2D expression on NK cells and CD8^+^ T cells, resulting in impaired cytotoxic effector functions [22], [23]. It has also been shown that leukemia/lymphoma T and B cells secrete NKG2D ligand-expressing exosomes with the ability to impair the cytotoxic potency of NK and T cells from healthy donors [24]. In a similar manner, NKG2D ligand-bearing exosomes have been shown to be actively released by placental explants and play a role in the immune evasion of the fetus [25]. Together, these data implicate NKG2D as a physiological target for exosome-mediated immune suppression.

Although there is accumulating evidence that tumor-derived exosomes are responsible for numerous immune-suppressive effects and tumor promotion [8], [22], [26], the role of patient-derived exosomes during PC progression has not been explored. PC is one of the most common malignancies, and the third leading cause of cancer death among men in the western world. Androgen ablation therapy is currently the standard treatment for advanced PC. However, the disease eventually progresses in most patients independently of circulating androgens; it is then termed castration-resistant PC (CRPC) and is at present incurable. In this study, we investigated whether exosomes derived from the serum or plasma of CRPC patients could affect NKG2D expression in immune effector cells. We found that circulating effector lymphocytes from patients with CRPC showed reduced NKG2D expression. Furthermore, exosomes derived from CRPC patients downregulated cell-surface NKG2D on NK cells and CD8^+^ T cells *in vitro*, similar to the immunosuppressive effects obtained by PC cell line (22Rv1) derived exosomes in this study. Our results indicate that secretion of exosomes may be a mechanism of tumor immune escape in PC.

## Materials and Methods

### Ethics statement

The study was approved by the local ethical review board of Umeå University (dnr 02-459) and all participants gave their written consent.

### Cell lines and blood samples

The 22Rv1, PC-3, LNCaP, and WPMY1 cell lines were purchased from the American Type Culture Collection (ATCC) and were maintained in RPMI 1640 GlutaMax (Invitrogen) supplemented with 10% ultracentrifuged fetal bovine serum (FBS; Invitrogen), 1 mM sodium pyruvate (Invitrogen), penicillin (100 U/ml; Invitrogen) and streptomycin (10 mg/ml; Invitrogen) at 37°C in a humified atmosphere with 5% CO_2_.

Blood samples were obtained (1) from patients with CRPC (defined as relapse after androgen-deprivation therapy) before initiation of treatment with chemotherapy (n = 18), and (2) from healthy male donors (n = 8).

### Exosome purification

Exosomes were isolated from culture supernatants, serum, or plasma by standard ultracentrifugation as previously described [5], [22], [24], [25], [27]. Briefly, supernatant fractions collected from 72-h culture and serum/plasma (diluted 1∶2 in PBS) were cleared of cells and debris by sequential centrifugations at 3,000 *g* for 30 min and 10,000 *g* for 35 min at 4°C. The pellet was discarded and the supernatant was passed through a 0.22-µm filter and ultracentrifuged at 110,000 *g* for 2 h. The exosome pellet was loaded on a 20–40% sucrose gradient and the ultracentrifugation step was repeated. The exosomes captured in the sucrose layer were collected and washed with PBS. The exosome pellet was resuspended in PBS and the protein concentration was determined using the BCA protein assay (Pierce).

### Isolation of lymphocytes

PBMCs from healthy men donors were isolated by gradient centrifugation on Lymphoprep (Nycomed). Lymphocytes were purified using magnetic-activated cell sorting (MACS) with microbeads (Miltenyi Biotec) according to the manufacturer's instructions. The purity of sorted populations was>95% (data not shown).

### Flow cytometry analysis of cell lines and lymphocytes

Adherent cells were harvested by treatment with 2 mM EDTA/PBS for 5 min and washed in FACS medium (PBS containing 3% bovine calf serum and 0.05% sodium azide). Lymphocytes, cultured 24–48 h with exosomes (10 µg/2×10^5^ healthy PMBCs) or untreated, were harvested washed in FACS medium and incubated with Human TruStain FcX (BioLegend) for 10 min at room temperature to block Fc receptors. Cells were stained with conjugated antibodies (Abs) on ice for 30 min in round-bottom 96-well plates. For intracellular staining, cells were fixed with 1% paraformaldehyde (PFA) for 30 min at room temperature and permeabilized with 0.5% saponin. After washing in FACS medium, the cells were stained with conjugated Abs. Staining was determined by flow cytometry (FACSCalibur; BD Biosciences FACS) and analyzed using CellQuest software (BD). Conjugated Abs used included anti-ULBP1-fluorescein isothiocyanate (FITC) (clone 170818), anti-ULBP2-phycoerythrin (PE) (clone 165903), anti-MICA/B-allophycocyanin (APC) (clone 159207), anti-CD3ε-FITC (clone UCHT1), and anti-NKG2D-Pe (clone 149810) (R&D Systems); anti-CD69-PE-Cy5 (clone FN50), anti-CD4-PE (clone RPA-T4), anti-CD16-APC (clone 3G8), anti-CD56-APC (clone B159), anti-CD8-APC (clone RPA-T8), and anti-CD95(Fas)-FITC (clone DX2) (BD Biosciences); and anti-CD178 (Fas-L)-PE (clone NOK-1) (BioLegend). Isotype-matched irrelevant Abs were used as negative controls.

### Flow cytometry analysis of exosomes

Isolated exosomes were coupled to surfactant-free 4-µm aldehyde/sulfate latex microbeads (Invitrogen), stained with conjugated Abs and analyzed by flow cytometry as previously described [25], [27], [28]. Briefly, the beads were coated with anti-CD63 (clone H5C6; BD Biosciences) and purified exosomes (10 µg) were added and incubated for 2 h at 4°C with end-to-end rotation. After washing, exosomes were stained with anti-ULBP1-FITC, anti-ULBP2-PE or anti-MICA/B-APC Abs. Isotype-matched irrelevant Abs were used as negative controls. Staining was determined by flow cytometry (FACSCalibur, BD Biosciences FACS) and analyzed using CellQuest software (BD).

### Western blot analysis

Proteins were solubilized using lysis buffer containing 0.5% NP40, 0.5% NaDOC, 0.1% SDS, 50 mM Tris-HCl (pH 7.5), 150 mM NaCl, 1 mM EDTA (pH 8.0), 1 mM NaF, and Complete Protease Inhibitor (Roche Diagnostics). Protein concentration was determined by the BCA protein assay (Pierce). Samples (4 µg protein) were separated by 10% SDS-PAGE under reducing conditions and subsequently transferred to PVDF membranes. Membranes were blocked in 5% milk before being incubated with primary antibody overnight at 4°C (diluted 1∶1,000 in 1% milk/PBST). The primary antibodies used were anti-CD63 (clone H5C6, BD Biosciences), anti-TSG101 (clone 4A10, Abcam), anti-GRP78 (ab53068, Abcam), and anti-PSMA (clone GCP-04, AbD Serotec). After washing in PBST, secondary antibodies (Dako, diluted 1∶20,000 in 2.5% milk) were applied and incubated for 1 h at RT. Protein expression was detected after extensive washing using the ECL Advanced Detection system (GE Healthcare).

### Electron microscopy of isolated prostate cancer-derived exosomes

Drops of 15 µl of isolated exosomes in PBS were put on 2% agarose with formvar/carbon-coated nickel grids on top and allowed to absorb for 5–10 min. The grids with adherent exosomes were fixed in 2% paraformaldehyd in PBS for 10 min followed by extensive washing in PBS. Negative contrast staining was performed with 1.9% methyl cellulose and 0.3% uranyl acetate for 10 min. The grids with negatively stained exosomes were dried and examined for morphology and size in a Zeiss EM 900 electrone microscope.

### Cytotoxicity assay

Cell-mediated cytotoxicity was performed using PBMCs from healthy donors as effector cells and the erythroid cell line K562, known to express NKG2D ligands, as target cells in a high effector: target ratio of 40∶1. Cell death was assessed by CytoTox 96 Non-Radioactive Cytotoxicity Assay (Promega), which measures cytoplasmic lactate dehydrogenase release. The assay was done according to the manufacturer's instructions. The K562 cells were incubated with isolated quiescent PBMCs at 37°C for 4 h to assess the baseline cytotoxic activity. In parallel, target and effector cells were incubated with native exosomes or Ab-blocked exosomes at a concentration of 40 µg/ml. For blocking of the NKG2D receptor anti-NKG2D blocking antibodies (clone 1D11, 10 µg/ml, BD Biosciences) were used. The NKG2D ligands were blocked with a cocktail of ULBP1–6 and MICA/B Abs (5 µg/ml). The specific lysis was calculated with a standard formula according to the manufacturer's instructions. Unstimulated PBMCs used as effector cells expressed <3% Fas (data not shown), assuring that the measured target cell lysis did not emanate from Fas-Fas-L interactions.

### Statistical analysis

The Mann-Whitney *U* and Kruskal-Wallis *H* non-parametric tests were used for comparisons between groups. A *P*-value<0.05 was considered statistically significant.

## Results

### Prostate cancer cells and exosomes express NKG2D ligands

**Figure 1:**
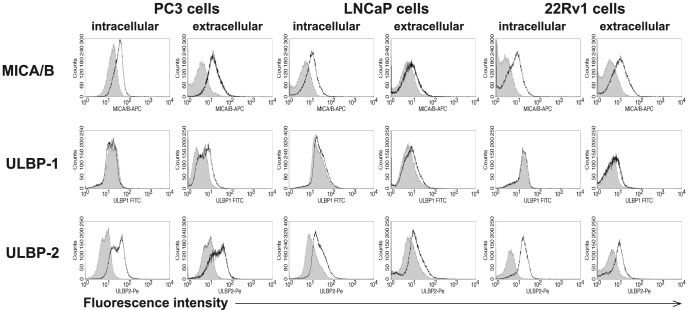
Expression of NKG2D ligands in prostate cancer cells. Human prostate tumor cell lines LNCaP, PC3, and 22Rv1 were stained with monoclonal antibodies (mAbs) specific for NKG2D ligands (ULBP-1, ULBP-2, and MICA/B) and analyzed by flow cytometry. Histograms show the mean fluorescence intensity (MFI) and the shaded histogram represents negative controls in which isotype-matched antibodies were used.

**Figure 2:**
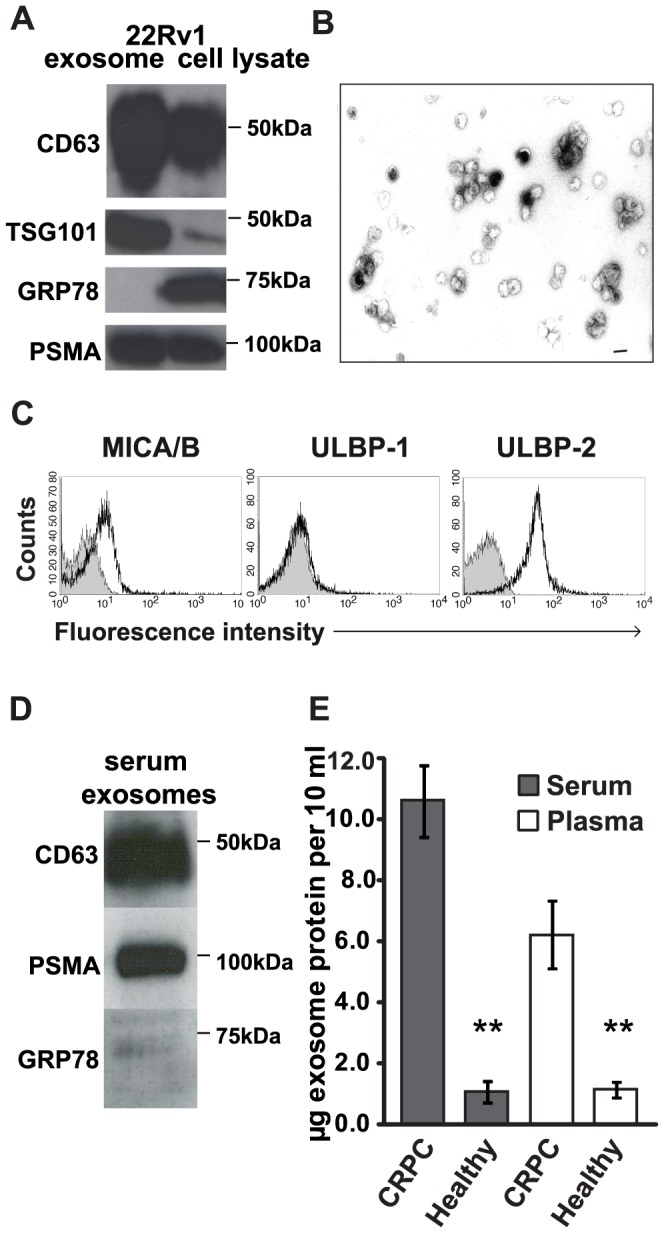
Characterization of prostate exosomes. (A) Western blot analyses of CD63, TSG101, GRP78 and PSMA in whole cell lysates of the 22Rv1 cell line or isolated exosomes from supernatants from this cell line. (B) Representative electron microscopic image of 22Rv1 exosomes, showing the typical cup-shaped morphology. Scale bar represents 100 nm. (C) Flow cytometry of 22Rv1 exosomes captured by CD63-coated latex microbeads and stained with mAbs to NKG2D ligands (ULBP-1, ULBP-2, and MICA/B). The histogram shows the mean fluorescence intensity (MFI) and the shaded histogram represents negative controls in which isotype-matched antibodies were used. (D) Representative western blot analysis of CD63, GRP78, and PSMA in exosomes isolated from serum of PC patients. (E) The total amount of exosomal protein per 10 ml of human fresh plasma or serum from healthy donors (n = 6–8 in each group) and CRPC patients (n = 17–18 in each group) was determined by BCA protein assay. ** *P*<0.01.

To study the expression of NKG2D ligands in PC, we first investigated ULBP1-2 and MICA/B expression in the human PC cell lines 22Rv1, PC-3, and LNCaP by flow cytometry. Variable NKG2D-ligand expression was observed in different PC lines, but all prostate tumor cells examined were positive for ULBP-2 and MICA/B ligands (Figure 1). 22Rv1 cells expressed high levels of ULBP-2 and MICA/B ligands, and they were therefore used for exosome isolation and in the experiments that followed (Figure 1). Exosomes from 22Rv1 supernatant were isolated using the standard method, sequential ultracentrifugation [5], [22], [25], [27]. To confirm the presence of exosomes and estimate the purity of isolation, the exosome fractions were evaluated by western blot and by negative-contrast staining and electron microscopy (EM). The western blot profile showed the presence of known exosome markers, including proteins CD63 and TSG101, and also the prostate-specific protein PSMA (Figure 2A). The absence of endoplasmic reticulum protein, GRP78, in the exosome preparations suggested high purity of the isolated exosome fractions (Figure 2A). EM analysis of the exosome fractions showed the uniform presence of microvesicles with the typical cup-shaped morphology of exosomes, measuring 50–100 nm in diameter (Figure 2B). To examine the expression of NKG2D ligands at the exosome surface, 22Rv1 exosomes were coupled to latex microbeads and analyzed by flow cytometry. 22Rv1 exosomes showed expression of MICA/B and ULBP-2 ligands, matching that of the 22Rv1 cells (Figure 2C).

### 22Rv1 exosomes selectively downregulate cell-surface NKG2D expression on NK and CD8^+^ T cells

**Figure 3:**
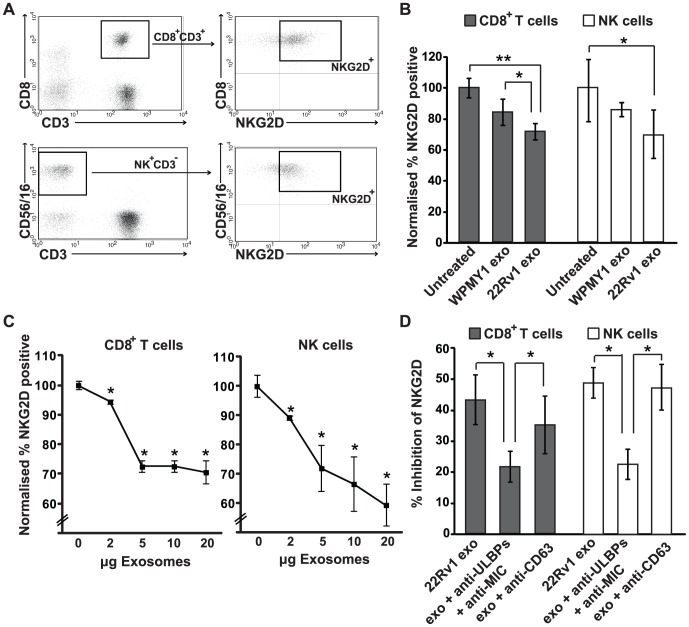
22Rv1 tumor exosomes downregulate cell-surface NKG2D expression on NK cells and CD8^+^ T cells. Purified 22Rv1 tumor exosomes or control fibroblast (WPMY1) exosomes were incubated for 24 h with healthy PMBCs, and flow cytometry was performed to determine the surface expression of NKG2D. (A) Representative dot-plots of flow cytometry gating scheme used to analyze NKG2D expression. (B) The bar graphs show the proportion of NKG2D-positive CD8^+^ T cells and NK cells relative to untreated cells (normalized to a value of 100). (C) The line graphs show the proportion of NKG2D-positive cells relative to untreated cells (normalized to a value of 100) when 22Rv1 exosomes were added at doses of 2–20 µg. (D) The percentage inhibition of NKG2D expression on CD8^+^ T cells and NK cells when 22Rv1 exosomes were treated with a mixture of anti-ULBP1-5 and MIC mAbs or anti-CD63 mAb. The graphs show mean ± SE (n = 8) from 2 independent experiments. * *P*<0.05; ** *P*<0.01.

Having demonstrated that 22Rv1 exosomes express MICA/B and ULBP-2 ligands, we next investigated whether these exosomes were able to downregulate cell-surface NKG2D expression, as reported for other NKG2D ligand-expressing exosomes [22], [25]. Peripheral blood mononuclear cells (PBMCs) were isolated from healthy donors and the NKG2D expression was analyzed after incubation with 22Rv1 exosomes. Expression of NKG2D was measured on CD8^+^ T cells and NK cells before and after 24 h or 48 h incubation with 22Rv1 exosomes, using flow cytometry (the gating scheme is shown in Figure 3A). Untreated PBMCs or PBMCs treated with fibroblast exosomes (WPMY1) were used as negative controls. CD8^+^ T cells and NK cells treated with 22Rv1 exosomes showed a significant reduction in cell-surface NKG2D expression after 24 h (Figure 3B). This downregulation was evident both as a 30% decrease in the proportion of NKG2D-positive cells (Figure 3B) and as a 30% reduction in the mean fluorescence intensity (MFI) compared to untreated PBMCs (data not shown). In contrast, fibroblast exosomes showed a negligible downregulation of NKG2D expression (Figure 3B and data not shown). This reduced NKG2D expression induced by 22Rv1 exosomes was NKG2D receptor-specific, since exosome treatment did not change the expression of CD3, CD8, CD56, CD16 or the activation marker CD69 (data not shown). After 48 h of incubation with 22Rv1 exosomes, there was a similar decrease of surface NKG2D expression in PBMCs as after 24 h of incubation (data not shown).

To examine whether the NKG2D receptor was internalized or merely masked on the cell surface by exosomes, PBMCs were fixed and permeablized, and intracellular expression of NKG2D was analyzed. PBMCs incubated with 22Rv1 exosomes had higher intracellular expression of NKG2D than untreated PBMCs (data not shown). Taken together, the extracellular and intracellular staining showed that total NKG2D expression was mainly intact, suggesting that the cell-surface downregulation of NKG2D by 22Rv1 exosomes was caused by internalization of the receptor (data not shown).

To determine whether down-modulation of NKG2D was exosome dose-dependent, 22Rv1 exosomes were added at doses of 2–20 µg per 2×10^5^ PBMCs. As illustrated in Figure 3C, NKG2D expression was significantly reduced in a dose-dependent manner. However, increasing the amount of exosomes above 5 µg had no significant additional effect on NKG2D expression, measured as the proportion of NKG2D-positive cells (Figure 3C). This pattern was also observed when we analyzed the level of NKG2D expression indicated by MFI (data not shown).

To establish whether exosomal NKG2D ligand expression was involved in the reduced expression of NKG2D, 22Rv1 exosomes were pretreated with a mixture of neutralizing ULBP- and MIC-specific monoclonal antibodies (mAbs). When the exosomes were preincubated with anti-ULBP and anti-MIC mAbs, the downregulation of NKG2D expression was significantly inhibited (Figure 3D). In contrast, preincubation of 22Rv1 exosomes with an anti-CD63 mAb, a known exosome protein, did not significantly affect the expression of NKG2D (Figure 3D). These results suggest that treatment of exosomes with anti-ULBP and anti-MIC was ligand-specific and that exosome expression of NKG2D ligands is partially responsible for downregulating the expression of NKG2D receptor. Together, these data demonstrate that prostate tumor 22Rv1 exosomes can selectively reduce the cell-surface expression of NKG2D receptor on NK cells and CD8^+^ T cells.

### 22Rv1-derived exosomes downregulate the NKG2D-dependent killing ability of PBMCs from healthy donors

**Figure 4:**
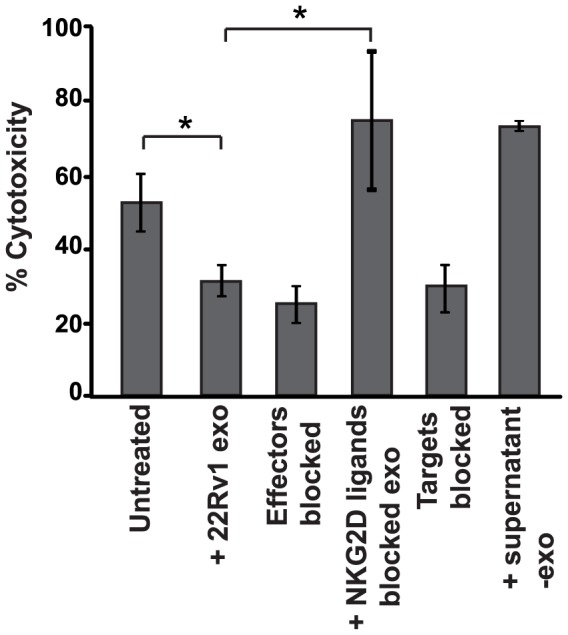
Effector lymphocyte cytotoxicity is impaired by 22Rv1 prostate tumor-derived exosomes. K562 target cells were incubated with PBMCs (effector cells), that were untreated, or treated with 22Rv1 exosomes, anti-NKG2D receptor-blocking mAb, or NKG2D-ligand blocked 22Rv1 exosomes. Additionally, target cells were blocked with a mixture of anti-ULBP1–5 and anti-MIC mAbs and the effect of 22Rv1 supernatant depleted from exosomes was also tested. The data represent mean values of 3 independent experiments ± SD. *P<0.05.

We then wanted to determine whether the selective downregulation of the NKG2D receptor by 22Rv1 exosomes on PBMCs affected their cytotoxic function. To assess impairment of effector cell function, an established CytoTox 96 Non-Radioactive Cytotoxicity Assay was used [24], [25]. PBMCs from healthy donors served as effector cells and the human NKG2D ligand-expressing, Fas-negative and MHC class I molecule-negative erythroleukemia cell line K562 [29] was used as target. To evaluate the effect of the PC exosomes under the most potent and stringent cytotoxic conditions, an effector-to-target ratio of 40∶1 was chosen for the cytotoxicity assay. As shown in Figure 4, the presence of 22Rv1 exosomes significantly reduced the lysis of the target cells. The level of exosome-mediated depression of cytotoxicity was comparable to that obtained by either blocking the receptor on effector cells or blocking the ligands on target cells. Furthermore, the cytotoxicity could be restored by specifically blocking the exosomes by pretreatment with anti-NKG2D ligand antibodies. These results suggest that downregulation of NKG2D levels by prostate tumor-derived exosomes has functional consequences, suppressing the *in vitro* killing ability of effector cells.

### Exosomal protein content is higher in CRPC patients than in healthy individuals

It was recently demonstrated that higher protein concentrations in plasma-derived exosomes were correlated with more aggressive melanoma disease [30]. To investigate whether the protein concentration of circulating exosomes in PC was exacerbated, we isolated exosomes from the plasma and serum of CRPC patients and healthy individuals using sequential ultracentrifugation. Using western blot, we confirmed the presence of the known exosome marker, CD63, and of the prostate-specific protein PSMA (Figure 2D). No expression of GRP78 protein indicated lack of contamination by endoplasmic reticulum-containing apoptotic cell debris fragments (Figure 2D). As illustrated in Figure 2E, exosome protein concentrations in both plasma and serum were significantly higher in CRPC patients than in healthy controls.

### Reduced NKG2D expression on circulating CD8^+^ T cells and NK cells in patients with CRPC

**Figure 5:**
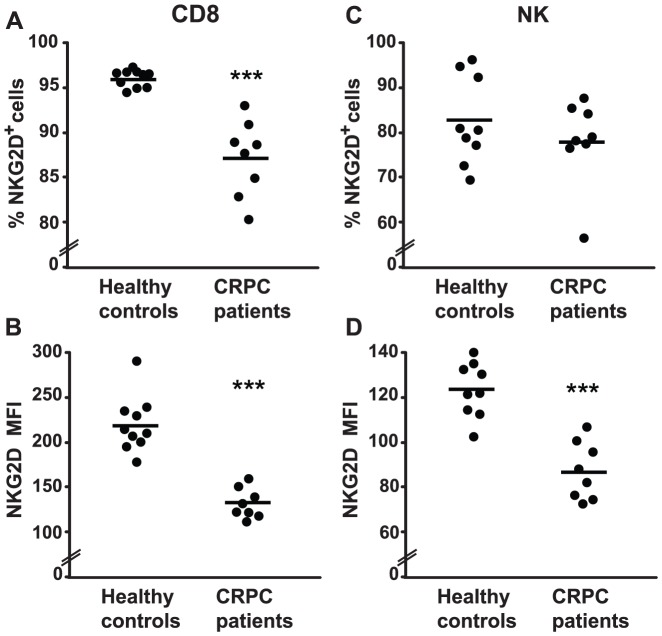
Surface expression of NKG2D is downregulated on circulating CD8^+^ T cells and NK cells from patients with CRPC. (A) and (B) NKG2D surface expression on circulating CD8^+^ T cells from healthy controls and CRPC patients was analyzed by flow cytometry. (C) and (D) NKG2D surface expression on circulating NK cells from healthy controls and CRPC patients. The graphs show the proportion of positive cells and the mean fluorescence intensity (MFI) of NKG2D as indicated. Each dot represents one individual and horizontal bars indicate mean values. *** *P*<0.001.

Our *in vitro* results demonstrating downregulation of surface NKG2D expression on NK cells and CD8^+^ T cells by 22Rv1 exosomes suggested to us that NKG2D expression in lymphocytes from PC patients could be impaired. We therefore examined NKG2D expression on circulating NK cells and CD8^+^ T cells in CRPC patients and in healthy controls by flow cytometry. NKG2D expression on CD8^+^ T cells from CRPC patients was significantly lower than in the healthy controls (Figure 5A and B). This NKG2D downregulation was also observed on NK cells from CRPC patients when we analyzed the MFI (Figure 5D), although the proportion of NKG2D-positive cells on circulating NK cells from CRPC patients was not statistically significantly different to that from healthy controls (Figure 5C).

### Serum-derived exosomes from patients with CRPC downregulate surface expression of NKG2D in effector lymphocytes

**Figure 6:**
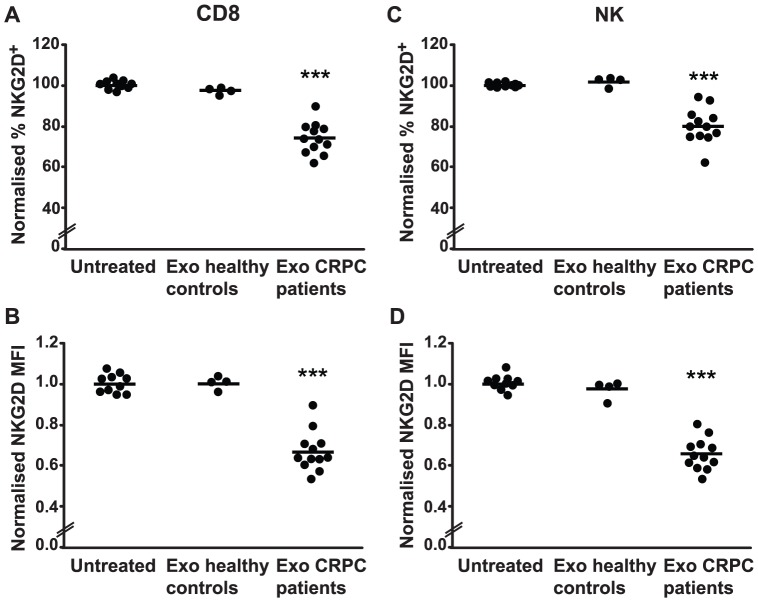
Exosomes isolated from patients with CRPC downregulate NKG2D expression. Serum-derived exosomes (2 µg) from healthy individuals or CRPC patients were incubated with healthy PMBCs, and flow cytometry was performed to determine NKG2D expression. (A) and (B) NKG2D surface expression on CD8^+^ T cells. (C) and (D) NKG2D surface expression on NK cells. The graphs show the proportion of positive cells and the mean fluorescence intensity (MFI) of NKG2D as indicated, relative to untreated PMBCs (normalized to a value of 100 and 1, respectively). Horizontal bars indicate mean values. *** *P*<0.001.

Having shown reduced NKG2D expression in effector lymphocytes from CRPC patients, we next examined whether this downregulation could be a consequence of circulating tumor exosomes. PBMCs from healthy donors were treated with exosomes isolated from the serum of CRPC patients or healthy controls. After 24 h of incubation, the NKG2D expression on NK cells and CD8^+^ T cells was assessed by flow cytometry. As illustrated in Figure 6, CRPC patient-derived exosomes induced an approximately 30% reduction in the level of NKG2D expression compared to exosomes isolated from the serum of healthy individuals. This downregulation of NKG2D measured by both MFI and by the proportion of NKG2D-positive cells was observed in CD8^+^ T cells (Figure 6A and B) and NK cells (Figure 6C and D) treated with serum exosomes from CRPC patients. In addition, we found a similar but not as profound downregulation of NKG2D expression (a 15% significant decrease in MFI) in effector lymphocytes when they were treated with plasma-derived exosomes from CRPC patients (data not shown). Together, these results support our hypothesis that tumor-derived exosomes downregulate NKG2D expression in PC patients and may be a possible mechanism of PC cells evading the immune system.

## Discussion

Tumor immune evasion is an important contributor to tumor progression, but the mechanisms are not completely understood. Recent studies have implicated tumor exosomes as immune-suppressive mediators [9], [10]. In this study, we found that circulating exosomes in PC patients selectively induce downregulation of the activating receptor NKG2D on NK cells and CD8^+^ T cells, and that this most likely impairs lymphocyte cytotoxic function and promote tumor immune escape.

NKG2D is a major activating cytotoxic immune receptor and its importance for immune surveillance of tumors is well-established [31]. Earlier studies have shown that various tumor cells express high levels of NKG2D ligands that after transplantation in mice caused an NKG2D-dependent tumor rejection [32], [33]. Furthermore, the degree of NKG2D ligand expression was found to correlate with the amount of T cell infiltrates in solid tumors [17]. However, despite the presence of MIC ligands on many progressing tumors [17], they still grow, indicating that there is inhibition of the MIC/NKG2D signaling in tumor cells. Recent evidence proposes tumor exosomes as important immunosuppressive microvesicles [22], [34]. Consistent with the data of Clayton *et al.* [22] who showed that exosomes from the human PC cell lines PC3 and DU145 express NKG2D ligands and downregulate NKG2D expression in effector lymphocytes, we show that 22Rv1-derived exosomes induced downregulation of NKG2D on NK and CD8^+^ T cells in a dose-dependent manner, leading to impaired cytotoxic function *in vitro*. Downregulation of NKG2D by 22Rv1 exosomes on NK cells and CD8^+^ T cells was partly dependent on exosomally expressed NKG2D ligands since blocking of ULBP1-5 and MICA/B on 22Rv1 exosomes enhanced NKG2D expression. However, NKG2D expression was not fully restored when exosomal NKG2D ligands were blocked. This could be due to insufficient blocking by the antibodies used or to other proteins present on the surface of 22Rv1 exosomes. Interestingly, TGFβ has been shown to be involved in NKG2D down-modulation and impairment of lymphocyte effector function [22], [35], [36]. It could be investigated further using the present experimental setup.

To determine whether reduced NKG2D expression could be physiologically relevant, we analyzed the *in vivo* expression of NKG2D in patients with CRPC. We found a significant decrease in surface NKG2D expression on circulating CD8^+^ T cells and NK cells from CRPC patients compared to healthy individuals. These data concur with a previous report by Wu *et al.* [19] who found that NKG2D expression was impaired on NK cells from PC patients. In addition, they showed a correlation between increased serum levels of sMIC, degree of impaired expression of NKG2D on NK cells, and grade of PC [19]. Importantly, we show here that prostate tumor-derived exosomes are most likely involved in downregulation of NKG2D in PC patients, as incubation of healthy lymphocytes with exosomes isolated from serum or plasma (data not shown) of CRPC patients triggered downregulation of NKG2D expression in effector lymphocytes. To our knowledge, this is the first demonstration of NKG2D downregulation by exosomes derived from PC patients. In line with this, other studies involving patients with progressive breast, lung, ovarian, or colon cancer have shown sMIC-induced downregulation of surface NKG2D expression on NK cells and/or cytotoxic T cells as a possible mechanism of tumor immune evasion [20], [37]. The secretion of tumor exosomes that reduce NKG2D surface expression, represents an alternative mechanism for suppressing lymphocyte functions to the one observed from MIC shedding due to matrix metalloprotease (MMP) cleavage [21], [38]. The logical question is whether functional differences in exosome- and sMIC-mediated downregulation of NKG2D exist. Comparison of functional differences of NKG2D receptor downregulation by exosomally carried and MMP-cleaved NKG2D ligands revealed that NKG2D ligands, expressed on exosomes, are far more potent as receptor down-modulators than the truncated MMP-cleaved soluble ligand [34], [39]. One explanation for the greater potency of exosome-mediated receptor downregulation is that, apart from preservation of the molecular structure and biological activity of the ligands, the exosomes can have several molecules of the same ligand and/or carry other NKG2D ligands simultaneously. Thus, they can serve as multipotent carriers of NKG2D ligands and are therefore able to impair the cytotoxic response to a greater extend [34], [39]. The presence of sMIC in the serum of cancer patients was proposed to be MMP-cleaved molecules [38]. However, the method of detection does not distinguish between MMP-cleaved and exosomally carried MIC molecules, as the protein moiety of MIC in the serum of cancer patients is a mixture of both. In this regard, it is important to note that the most abundantly expressed MIC protein, MICA*008, is exclusively released on exosomes [34].

Further evidence for how impairment of NKG2D expression might contribute to tumor cells escaping NK cell and cytotoxic T cell immune surveillance comes from the analysis of NKG2D-deficient mice [40]. In NKG2D-/- TRAMP mice, a transgenic model of prostate adenocarcinoma [41], the absence of NKG2D resulted in enhanced formation of aggressive tumors [40]. Similarly, NKG2D-deficient Eμ-myc mice, a transgenic model of B cell lymphoma, developed lymphomas substantially earlier than wild-type Eμ-myc mice [40]. The effect on tumor incidence was probably a direct effect of missing NKG2D expression, as NK cells and other cells developed normally in NKG2D-deficient mice [40].

However, NKG2D downregulation is not the sole mediator of exosomally-induced immune escape. In line with recent evidence of Fas-L-expressing tumor exosomes [7], [26], [42], [43], we have observed that secreted exosomes from human prostate 22Rv1 cells express Fas-L and induce apoptosis in activated Fas-expressing CD8^+^ T cells (data not shown). These findings emphasize tumor exosomes as pluripotent suppressors of the immune response.

In conclusion, our findings highlight exosomes derived from PC patients as agents responsible for downregulation of NKG2D, which may subsequently facilitate immune escape. Still, further studies are needed to establish unequivocally that secretion of PC-derived exosomes is a major immune-suppressive mechanism contributing to tumor progression. Studies of tumor exosomes may provide novel immunotherapy strategies for improvement of the function of effector lymphocytes.
